# Amygdalar glutamatergic neuronal systems play a key role on the hibernating state of hamsters

**DOI:** 10.1186/1471-2202-12-10

**Published:** 2011-01-20

**Authors:** Raffaella Alò, Ennio Avolio, Antonio Carelli, Rosa Maria Facciolo, Marcello Canonaco

**Affiliations:** 1 Comparative Neuroanatomy Laboratory of Ecology Department, University of Calabria, Ponte Pietro Bucci, 87030 Arcavacata di Rende, Cosenza, Italy

## Abstract

**Background:**

Excitatory transmitting mechanisms are proving to play a critical role on neuronal homeostasis conditions of facultative hibernators such as the Syrian golden hamster. Indeed works have shown that the glutamatergic system of the main olfactory brain station (amygdala) is capable of controlling thermoregulatory responses, which are considered vital for the different hibernating states. In the present study the role of amygdalar glutamatergic circuits on non-hibernating (NHIB) and hibernating (HIB) hamsters were assessed on drinking stimuli and subsequently compared to expression variations of some glutamatergic subtype mRNA levels in limbic areas. For this study the two major glutamatergic antagonists and namely that of N-methyl-D-aspartate receptor (NMDAR), *3-(+)-2-carboxypiperazin-4-yl-propyl-1-phosphonate *(CPP) plus that of the acid α-amine-3-hydroxy-5-metil-4-isoxazol-propionic receptor (AMPAR) site, *cyano-7-nitro-quinoxaline-2,3-dione *(CNQX) were infused into the basolateral amygdala nucleus. Attempts were made to establish the type of effects evoked by amygdalar glutamatergic cross-talking processes during drinking stimuli, a response that may corroborate their major role at least during some stages of this physiological activity in hibernators.

**Results:**

From the behavioral results it appears that the two glutamatergic compounds exerted distinct effects. In the first case local infusion of basolateral complexes (BLA) with NMDAR antagonist caused very great (p < 0.001) drinking rhythms while moderately increased feeding (p < 0.05) responses during arousal with respect to moderately increased drinking levels in euthermics. Conversely, treatment with CNQX did not modify drinking rhythms and so animals spent more time executing exploratory behaviors. These same antagonists accounted for altered glutamatergic transcription activities as displayed by greatly reduced GluR1, NR1 and GluR2 levels in hippocampus, ventromedial hypothalamic nucleus (VMN) and amygdala, respectively, plus a great (p < 0.01) up-regulation of GluR2 in VMN of hibernators.

**Conclusion:**

We conclude that predominant drinking events evoked by glutamatergic mechanisms, in the presence of prevalently down regulated levels of NR1/2A of some telencephalic and hypothalamic areas appear to constitute an important neuronal switch at least during arousal stage of hibernation. The establishment of the type of glutamatergic subtypes that are linked to successful hibernating states, via drinking stimuli, may have useful bearings toward sleeping disorders.

## Background

The participation of the amygdala (AMY) in a growing number of neuroprotective functions is making this telencephalic limbic area a key site for many physiological activities such as hibernation. AMY is composed of spatially contiguous plus anatomically interconnected nuclei i.e. basolateral (BLA) and cortico-medial complexes [1]. It influences a number of emotional and mnemonic features, especially through extensive visceral (hypothalamus and olfactory lobes) and autonomic-somatomotor connections [2-4]. Interestingly, these connections seem to control the induction of rapid eye movement (REM) as well as the awaking state via the suppression of glutamate (Glu) excitatory signals [5,6]. At date, satiety states controlled by BLA plus its anatomical connections to the hypothalamus (HTH), hippocampus (HIP) and cortex (COR) have largely pointed to HIP as a main limbic switch controlling feeding habits [1]. This type of control seems to strongly rely on some classes of glutamatergic receptor subunits such as N-methyl-D-aspartate receptor (NMDAR) subtypes (NR1, NR2A-D, NR3) of the ionotropic Glu family, which have shown to modulate a large amount of activities ranging from development, synaptic plasticity to mnemonic neuronal processes events [7-10]. In the case of the other class of ionotropic Glu receptor, the α-amino-3-hydroxi-5-methyl-4 isoxazole receptor (AMPAR) is a homo- or hetero-oligomeric site composed of GluRA-GluRD (or GluR1-GluR4) subunits, which are responsible for the majority of fast excitatory synaptic transmission. In addition, recent reports have demonstrated that these subtypes are not only actively involved with plasticity activities of learning and memory processes, but also with the induction of spontaneous locomotor behaviors in rats [11,12].

It is largely known that the glutamatergic neuronal system is composed of two principal classes of receptor complexes. A first class consisting of NMDAR subtypes (NR1, NR2A-D, NR3) of the ionotropic Glu family is involved in a large amount of activities ranging from mnemonic processes to excitotoxicity events occurring during cerebral ischemia and neurodegenerative disorders such as Parkinson's and Huntington's diseases as well as epilepsy and neuropathic pain disorders [7-10]. The AMPAR family is responsible for the majority of fast excitatory synaptic transmission activities [13]. Previously, studies have highlighted a key NR1,2 blocking type of event on some neurotransmission activities while NR3 seems to be more specific for the excitatory events of other receptor systems such as glycine receptor [13,14]. In particular, NMDAR dynamic membrane trafficking seems to exert a critical role on feeding habits of the Arctic ground squirrel very probably through the elicitation of taste centers during the entrance stage of hibernation [15-17].

For this study hibernation, which is a well known physiological phenomenon leading to hypothermia and thus the survival of animals under extraordinary stressful conditions constitutes a major and valuable experimental paradigm [18]. Indeed during the different states of this physiological condition a neuroprotective program with adaptive homeostatic mechanisms is activated [19,20]. Interestingly, the 8 day hibernating cycle features a torpor bout that is interrupted by brief spontaneous pre-arousal sessions followed by an arousal period (last four days of hibernation) so that the different states are characterized by a decrease in body temperature with short periods of inter-bout euthermy [21,22]. It is noteworthy that our rodent model i.e. the Syrian golden hamster (*Mesocricetus auratus*) is a permissive hibernating rodent characterized by peculiar brain vascular properties and this may have strong bearings on the necessity of maintaining a constant hypothalamic osmoregulation state throughout the wake-sleep cycle of rodents [15,23]. Hence on the basis of the above information it was the aim of the present study to define the type of BLA-dependent Glu neurochemical mechanisms responsible for overall molecular cross-talking mechanisms operating on both drinking plus feeding behaviors of hibernators [24]. In addition the behavioral effects were also related to expression variations of NMDAR and AMPAR subtypes in key limbic areas such as AMY, HTH, HIP and COR that may exert a key switching ON/OFF role of hibernation. These results could be a good starting point for corroborating the precise role of BLA on water consumption with interesting bearings regarding their neurocircuitry activities of some limbic areas occurring in occasion of sleeping episodes since the increase of extracellular fluid appears to exert a stimulatory effect, above all during the arousal state [15,22,24,25].

## Methods

### Animals and stereotaxic surgery

**Figure 1:**
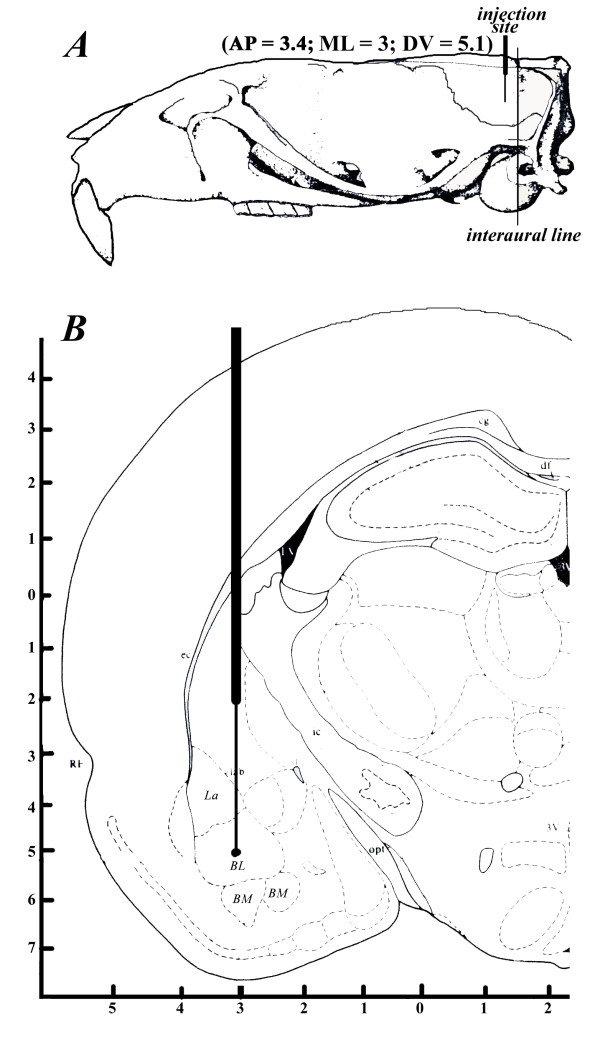
**Dorsal and lateral views of the skull of a 150 gr male Syrian golden hamsters**. The position of the guiding canula between the horizontal plane passing through bregma and lambda and that of the interaural line is shown (A) along with this position leading to the target site, i.e. BLA complex (B).

For this study adult male Syrian golden hamsters (Charles River, Como Italy; n = 32) weighing 120-150 gr were anesthesized with a dose (0.2 gr/Kg body weight) of urethane and underwent drug infusion using stereotaxic methods (Figure 1A-B) by implanting a guide cannulae unilaterally in the left BLA (AP+3.4 mm, ML+3.0 mm from lambda, DV-5.1 mm to the skull surface) that was left there for the entire treatment session. Afterwards, euthermic hamsters received one microinjection of 1 μl containing either a final concentration of 7.93 nM CPP (*3-(+)-2-carboxypiperazin-4-yl-propyl-1-phosphonate*) an NMDAR antagonist (n = 8), or 36.2 nM of CNQX (*cyano-7-nitro-quinoxaline-2,3-dione*), an AMPA receptor (AMPAR) non-NMDAR antagonist (n = 8) dissolved in saline at the beginning of their hibernating cycle. This treatment was conducted every morning for the entire duration of hibernation (8 days) 30 minutes before behavioral observations. During each treatment session the animals were transferred from their cage to another neutral cage (one/cage) where they received the above antagonists and checked to establish the role of these glutamatergic agents on feeding and drinking frequencies for both the non-hibernating (euthermic) and hibernating states with respect to controls (n = 8) that only received 1 μl saline solution. The concentrations and volumes were chosen on the basis of their effects towards motor behaviors reported for other rodents such as rats as well as on the absence of drug spreading effects to other adjacent areas of BLA [26]. The inner cannula was left in place for an additional 60 s to allow diffusion of the solution and to reduce the possibility of reflux. Other animals that received a saline solution were either assigned to euthermic (n = 4) or to hibernating (n = 4) conditions for future RNA extraction procedures required for the production of NR1/2 primers. Five days after having implanted the cannula, hamsters were tested and then one day prior to the onset of behavioral testing they received a first microinjection of drugs, which coincided with the same time interval used previously by others [27]. At the end of behavioral observation the animals were euthanized with chloroform and were injected with 1 μl of 1% methylene blue solution in order to verify the injection site of the left BLA. Subsequently, these animals were decapitated and their brain were removed, blocked and prepared for coronal sections (12 μm). In this study only the data obtained from animals that had the cannula correctly placed in this AMY area were collected and taken into consideration.

Animal maintenance and all experimental procedures were carried out in accordance with Guide for Care and Use of Laboratory Animals issued by the European Communities Council Directive of 24 November 1986 (86/609/EEC). Efforts were made to minimize animal suffering and reduce the number of specimens used.

### Motor and feeding behaviors

For the present study our permissive facultative hibernating hamster, which regularly undergoes bouts of torpor enabled us to examine frequent neuronal changes evoked throughout the entire 8 days hibernating cycle [28]. In a first part, euthermic animals were maintained on a photoperiod of 12-h/12-h light/dark cycle (lights on at 0700 h) at a temperature of 25°C. Other hamsters (hibernators) were maintained on a 8:16 h light/dark cycle at a temperature of approximately 8°C, due to its permissive hibernating capability allowing them to frequently enter and exit from this physiological state [29]. Both animal groups either received a NMDAR (n = 8) or AMPAR (n = 8) antagonist and compared to controls (n = 8) of their respective physiological states prior to behavioral analyses. In this case, three observation sessions consisting of morning (10 a.m.), early (2 p.m.) and late (6 p.m.) afternoon were carried out in which drinking and feeding behaviors were estimated during two 1/2 h observations for each session. The amount of food and water, given *ad libitum *that was consumed during the "lights on cycle" of this period was evaluated.

In the behavioral session, the following conditions were quantified:

1. Food and water consumption were determined for both euthermic phase and hibernating bouts by estimating the daily amount of rodent chow pellets and water consumed in an interval of four-one half hour observations conducted during the above indicated times. These values were based on previously weighed pellets per day for the entire torpor stage (3^rd ^to 5^th ^day) of the hibernators plus the registration of the precise water volume given to all animals and subtracting the quantities of chow and water not consumed (controlling that no water leakage occurred).

2. The time spent executing feeding and drinking behaviors were also monitored with respect to the typical motor behaviors, i.e. raising of back, circling during the total hibernation period starting at day 0 and continuing up to the entire 8 day cycle. In this same session the volume of water consumed during the last four days of hibernation, which coincide with the two days prior and to the two days of arousal was estimated according to the same observation modality as above. Moreover, drinking rhythms were compared to behavioral motor activities during the same above observation interval of the last four days of hibernation. From such evaluations, water volume estimated during the entire behavioral observations turned out to be a useful indicator to further establish the percentage of water intake during different days of torpor state.

During the entire behavioral sessions the time spent by all animals in the different motor behaviors plus the amount of chow pellets and water consumed, along with the amount being lost in the litter was recorded during the three different observation sessions (morning, noon and afternoon) with a NV-VS7 Panasonic video camera (Tokyo, Japan).

### Molecular characterization

At the end of the behavioral observations (6 p.m.) that coincided with the last of the three and eight treatment doses for euthermics and hibernators, respectively, the brain was removed and stored at -40°C for neuroanatomic and molecular studies. For verification purposes, a series of 20 μm sections deriving from euthermics were stained with thionin in order to establish the position of the cannula. Subsequently, the sequence of NR1 and NR2A was determined from the total RNA extracted from the whole brain of euthermic hamsters (n = 4) using TRI reagent dissolved in DEPC-water. Reverse transcription (RT) reaction was performed using 2 μg total RNA with RETROscript kit at 44°C for 1 h after template denaturation at 75°C for 3 min. Then PCR was carried out on the different dilutions of cDNA obtained by RT using the following coupled primers as described by Chowanadisai et al. [29]: NR1 forward primer (5'ccaggctgtgcgggacaac a3') and NR1 reverse primer (5'tggaggccagggtggaggtg3') binding to position 2419-2438 and 2933-2914, respectively, of *Rattus norvegicus *sequence (NM_ 017010); NR2A forward primer (5'ccacactgcccatgaacaggaa cc3') and NR2A reverse primer (5'ggctcccatacaagttgccc tcca3') binding to position 3488-3511 and 3982-3959, respectively, of *Rattus norvegicus *sequence **(**NM_ 012573). PCR was performed with the following programs: NR1 - initial incubation at 94°C for 2', amplification was carried out for 33 cycles at 94°C for 30'', at 59°C for 30'', at 72°C for 1' while the last primer extension step was at 72°C for 2'; NR2A - initial incubation at 94°C for 2', amplication phase for 34 cycles at 94°C for 30'', 55°C for 30'', 72°C for 1' and the last extension step at 72°C for 2'. Subsequently, amplified products (approximately 10 μl volume) were separated on 1.5% agarose gel and visualized by ethidium-bromide staining under UV illumination.

### Transcription activity of NMDAR and AMPAR subtypes

Synthetic oligonucleotide probes were designed from the sequences of the above PCR products: the antisense probes were complementary to the encoding sequences of bp 2367-2412 for NR1 and bp 3644-3689 for NR2A of the mature protein for NR1 (5'agtaaccacgccctcagcgtaagtactatggcttgggtacagaag3') and NR2A (5'attgtaaagtggcctgagtaggtgg gcagattagaaaggcaactt3'). For GluR1 and GluR2 probes, due to comparable results of mice probes being applied to other animal models, neuronal expression capacities of these AMPAR subtypes were also done using probes specific for mouse [30]. The antisense probes were complementary to the encoding sequences of bp 1959-2003 for GluR1 and bp 1919-1963 for GluR2 of the mature protein for GluR1 (5'gtcactggttgtctgatctcgtccttcttcaaactcttcactgtg3') and GluR2 (5'ttcactactttgtgtttctcttcac tttcaaattcctcagtgtg3'), respectively.

Afterwards, *in situ *hybridization was carried out on hamster brain sections using antisense and sense probes labeled by 3'-tailing with digoxigenin-11-dUTP (DIG) according to the instructions supplied by DIG oligonucleotide tailing kit. The preparation of the probe was done via its incubation at 37°C for 30 min and stopped with 0.2 M EDTA pH 8.0. Probe concentration was determined by quantification against known standards on Hybond N^+ ^filters. Then posterior brain sections (10 μm) of euthermic (n = 8) and hibernating (n = 8) hamsters treated with antagonists of NMDAR and AMPAR with respect to their controls (n = 8) were incubated with 100 ng of antisense probe in 100 μl of hybridization solution for overnight *in situ *hybridization at 50°C in a humidified chamber as previously reported [31]. Nonspecific hybridization was obtained on slides incubated with the sense probe. For immunological detection, sections were cover-slipped for 45' with PBS buffer containing 2% normal sheep serum and 0.3% Triton X100. Then an anti-digoxigenin alkaline phosphatase antibody 1:100 was added for 2 h at room temperature and the alkaline phosphatase color reaction buffer (NBT/BCIP) was added to sections and incubated for 72 h in a humidified dark chamber. At the end of this time, reaction was blocked by a solution of Tris-HCl 100 nM plus EDTA 1 mM. Neuronal hybridization signals of these posterior brain areas that include AMY, HTH, HIP and COR areas were viewed at a bright-field Dialux EB 20 microscope (Leitz) under a phase contrast objective (40X). Transcriptional activity was evaluated using a Panasonic Telecamera (Canon Objective Lens FD 50 mm, 1:3.5) attached to a Macintosh computer-assisted image analyzer system by running an Image software of NIH.

#### Statistical analysis

The changes induced by the two NMDAR and AMPAR antagonists on feeding and drinking behaviors plus on motor behaviors were evaluated by a two-way repeated measures for Analysis Of Variance (ANOVA) and differences were established by using Scheffe's *post hoc *comparison test or Newman-Keul's multiple range *post hoc *test when there was a significant *p*-value ≤ 0.05. The mRNA transcript levels (mean ± s.e.m.) of NMDAR (NR1 + NR2A) and of AMPAR (GluR1 + GluR2) expressing neurons were compared among non-hibernating and hibernating hamsters treated with Glu antagonists with respect to their controls, by using ANOVA followed by Newman-Keul's multiple range *post hoc *test when p value ≤ 0.05.

## Results

### Feeding and drinking behaviors

**Figure 2:**
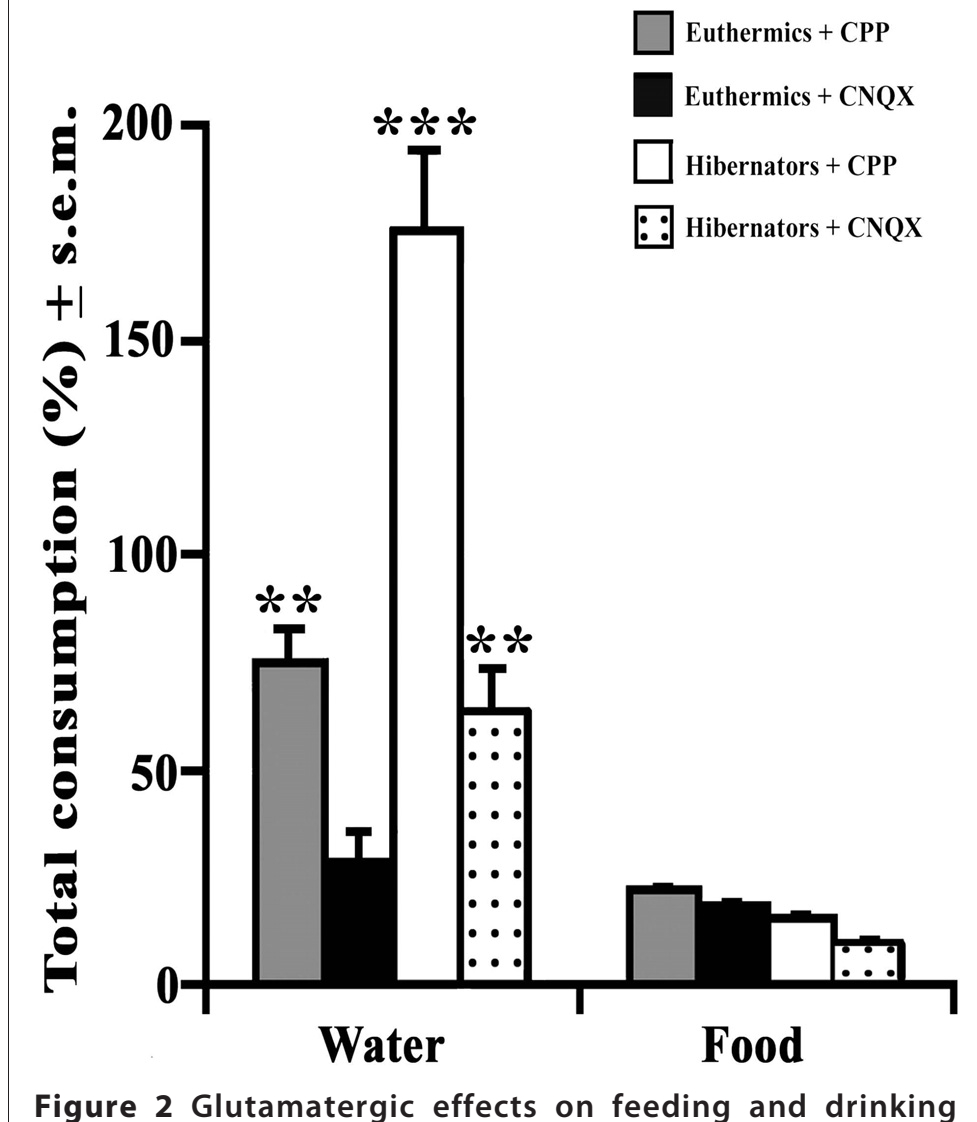
**Glutamatergic effects on feeding and drinking behaviors of euthermic and hibernating hamsters**. Glutamatergic-dependent activities were induced by locally administering most effective concentrations of NMDA (CPP; 7.93 nM, grey and white squares) and AMPA antagonists (CNQX; 36.2 nM, black and dotted squares) into BLA of both euthermic (grey and black squares) and hibernating (white and dotted squares) adult Syrian golden hamsters (n = 16) with respect to NMDAR and AMPAR controls (n = 8) using stereotaxic methods. Total feeding (g) and drinking volume (ml) percentages (mean ± s.e.m.) were calculated with respect to controls that only received a saline solution. Data were analyzed by two-way ANOVA followed by Newman-Keuls *post-hoc *test when a p-value ≤ 0.05. *p < 0.05; **p < 0.01; ***p < 0.001. Comparisons were handled on both the two physiological states and treatment groups that are numerically indicated above histograms.

The BLA of hibernating and euthermic hamsters infused with the two major glutamatergic antagonists (CPP and CNQX) accounted for elevated water consumption (from +29 to +170%) with respect to controls (Figure 2). In the case of feeding behaviors neither antagonists seemed to produce significantly evident changes. Regarding water consumption, it seemed that both antagonists were responsible for a significantly evident rising trend (F_(5,19) _= 9.84; p < 0.01) of drinking rhythm. Surprisingly, euthermics that were treated with either NMDAR or AMPAR antagonists and compared to controls prevalently showed notable drinking rhythms especially for CPP-treated hamsters in which a great (p < 0.01) amount of water consumption (+78%) was detected (Figure 2). The drinking effects of this NMDAR antagonist turned out to be more conspicuous in hibernators as displayed by a very great (p < 0.001) amount of water volume (+175%) being consumed with respect to controls. On the other hand, AMPAR antagonist (Figure 2) only accounted for a great amount of water consumed (+65%) during the torpor state.

**Figure 3:**
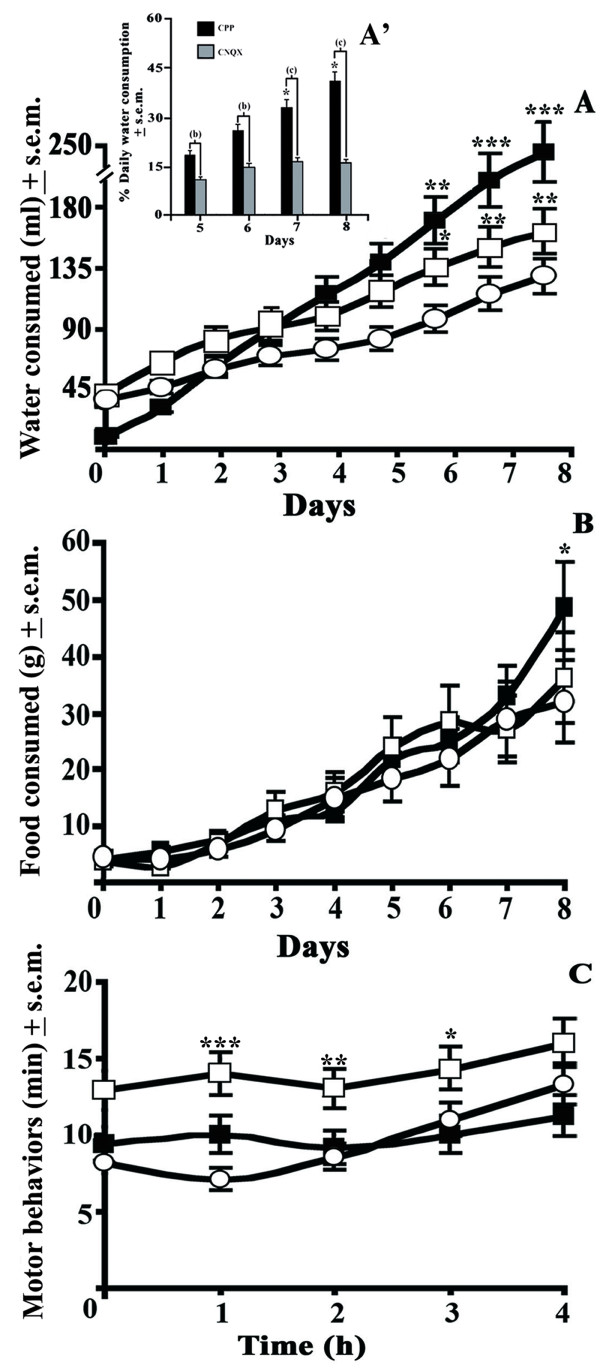
**Glutamatergic effects on feeding and water consumption plus on overall behavioral activity during hibernation**. The influences of the same treatment modality reported in Figure 1 were assessed on (A) water (ml) and (B) food (g) consumption of hamsters for the entire 8 days hibernating session with the first day prior to it being considered as a negative control. These effects were also compared to (C) overall motor behavior of the hamster, this time during a 4 h interval prior to arousal. At the same time the role of CPP (black square) and CNQX (white square) on total water consumption with respect to controls (white circle) were compared during the latter part of this physiological state and precisely the last four days of hibernation (Figure 3A'). The variations induced by both CPP- and CNQX-treated animals were compared in the same manner to that used in Figure 2.

When the different motor behavioral activities over the entire hibernating cycle were assessed, it was still the drinking rhythm that continued to display a significantly evident rising trend (F_(3,9) _= 27.40; p < 0.01) especially during the latter part of the hibernating cycle (Figure 3). It is worthy to note that the possibility of measuring water consumption during the inter-bout periods of torpor are mainly due to this permissive hibernator being able to frequently wake up. Indeed, it was the end of the torpor state, coinciding with the beginning of arousal stage, that turned out to be critical for the consumption of water as shown by a greater drinking rhythm (+80%) on day 6, which continued to be of a very great (>+134% <+195%) entity between day 7 and 8, respectively, for hamsters treated with CPP (Figure 3A) as compared to their controls. Conversely, CNQX-treated animals provided a moderately greater consumption on day 6, which only rose to a greater quantity (>+62% <+73%) for the same two latter days, with respect to their controls. Interestingly the role of the NMDAR antagonist on drinking stimuli turned our to be of a greater entity when we compared the influence of both antagonists on this physiological drive during the last four days of hibernation. In this case it was the fifth and sixth day of torpor that hamsters treated with CPP continued to display a great influence on drinking episodes (+62%) with respect to CNQX-treated animals and subsequently such an effect abruptly rose as shown by greater CPP-related drinking variations on days 7 (+115%) and 8 (+205%; Figure 3A'). Conversely, local infusion of glutamatergic antagonists did not modify eating activities, aside CPP having accounted for a moderate increase (+50%) of food consumption on the 8^th ^of hibernation (Figure 3B). However, during the arousal state and in a specific manner the four hours which precede the arousal state, hamsters treated with CNQX appeared to dedicate more time to motor behaviors (Figure 3C) and less time to drinking episodes with respect to the very amount of time hamsters spent drinking water when treated with CPP. This feature is particularly evident during the early part of the arousal stage in which the AMPA antagonist accounted for a very great amount of time (105%) involved with all other motor activities (evaluated in the present study) and not drinking while the motor activities diminished with time until the final 8^th ^day of arousal.

### Molecular characterization

In order to analyze the expression of NR1 and NR2A subunits in hamster brain, RT-PCR methods were performed by using a pair of primers that in the rat brain featured only one amplification product at 500 bp, which is similar to that obtained in the euthermic adult hamster for both subunits [32]. In the case of GluR1 and GluR2 probes, those designed from mice sequences, due to comparable results with other animal models were used for the present study.

#### Influence of CPP and CNQX on AMPAR plus NMDAR expression differences

**Figure 4:**
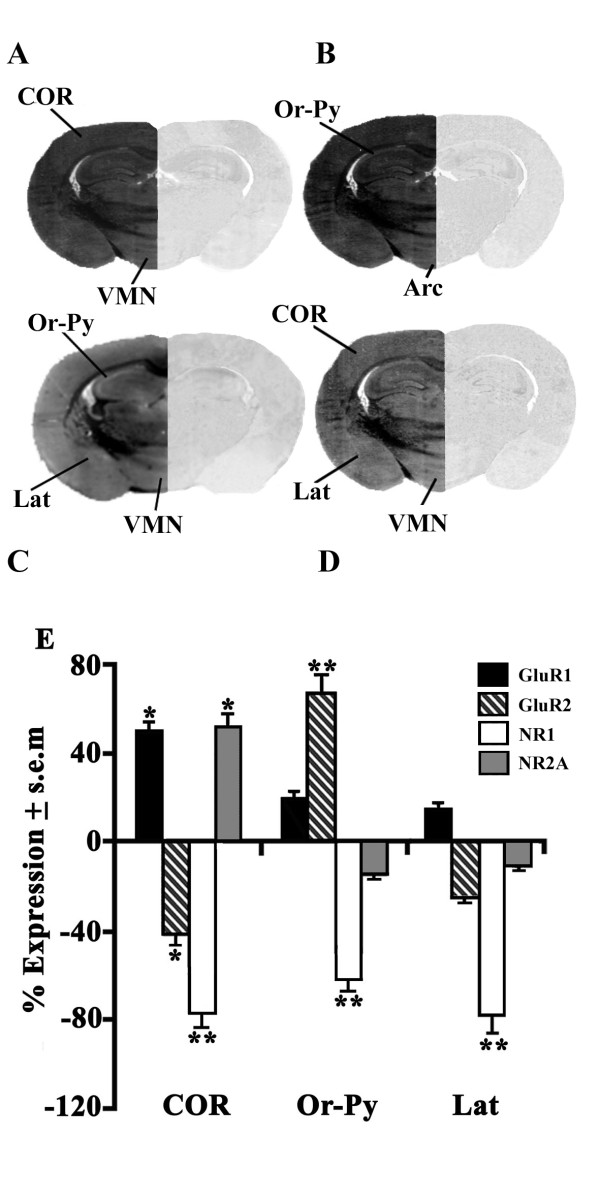
**Brain expression of AMPAR and NMDAR mRNAs in CPP-treated euthermics**. Transcription expression of antisense (left half section) and sense (right half section) probes of GluR1 (A), GluR2 (B), NR1 (C) and NR2A (D) showed an heterogeneous distribution pattern for all brain regions of euthermic hamsters as observed in representative posterior brain regions. (E) The expression percentage (% ± s.e.m.) of GluR1 (black), GluR2 (black-white stripes), NR1 (white) and NR2A (grey) mRNAs of CPP-treated hamsters (n = 4) were compared to their controls (n = 4). Values were analyzed by a two-way ANOVA followed by Newman-Keul's Multiple Range Test when p < 0.05. *p < 0.05; **p < 0.01; ***p < 0.001. Abbreviations: Arc, arcuate hypothalamus nucleus; Ce, amygdala central nucleus; COR, cortex layer; GD, hippocampal dentate gyrus; Lat, amygdala lateral nucleus; Or-Py, oriens-pyramidalis layer of the hippocampus; VMN, ventromedial nucleus of the hypothalamus.

**Figure 5:**
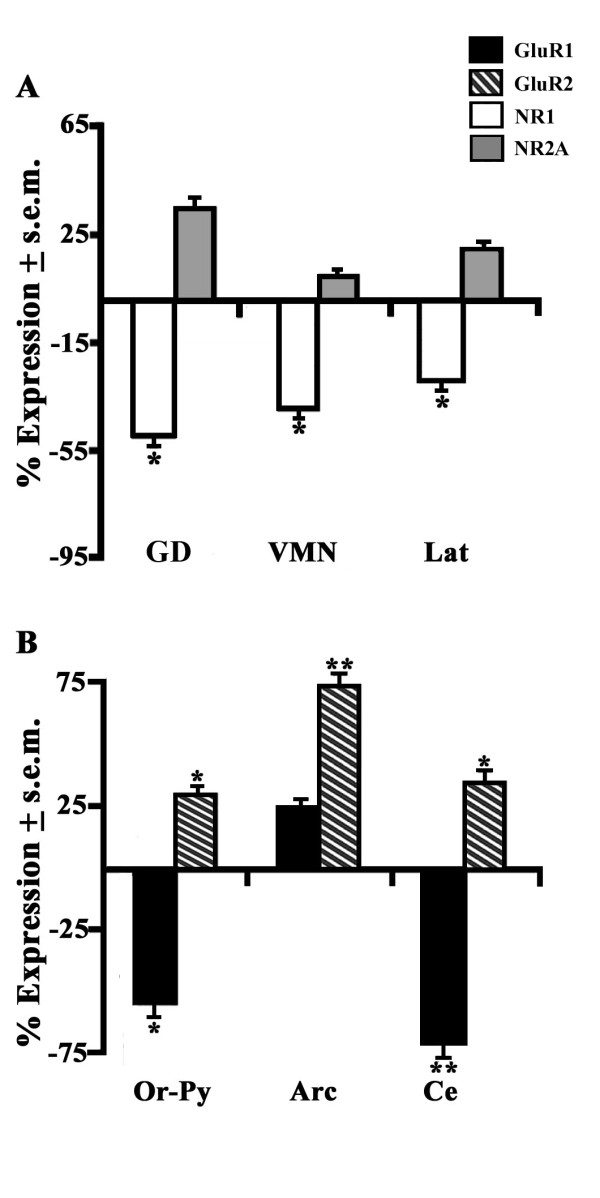
**Brain expression of NMDAR and AMPAR mRNAs in CPP-treated hamsters during torpor state**. Effects of CPP (n = 4) on expression percentage (% ± s.e.m.) of (A) NR1 (white) and NR2A (grey) plus of (B) GluR1 (black) and GluR2 (black-white stripes) in some telencephalic and hypothalamic areas of hibernating hamsters were compared to controls (n = 4). AMPAR and NMDAR mRNA variations induced by CPP during torpor state were compared in the same manner to that of Figure 4. For abbreviations also check this Figure.

The molecular analysis conducted by *in situ *hybridization showed an evident distribution pattern of AMPAR (GluR1 and GluR2) and NMDAR (NR1 and NR2A) expressing neurons as illustrated by the heterogenous distribution pattern of these subtypes in representative posterior forebrain sections of euthermic hamsters (Figure 4A-D). In a first case, hamsters that received a local infusion of CPP supplied moderately high and strong densities of GluR1 in cortex (COR, +48%) and of GluR2 in the oriens-pyramidalis HIP layer (Or-Py, +67%), respectively (Figure 4E). This NMDAR antagonist also continued to be responsible, aside for the moderately elevated up-regulation of NR2A in COR (+54%), for a strong down-regulation of NR1 mRNA levels in some AMY nucleus and namely the lateral amygdala nucleus (Lat; -78%) along with COR (-71%) and Or-Py (-60%). As far as the effects of the NMDAR antagonist on the hibernating hamster are concerned, it accounted for a moderately reduced activity of NR1 expressing neurons in some HTH nuclei such as the ventromedial nucleus (VMN, -40%; Figure 5A). Moreover, in these same brain areas such as Lat (-35%) as well as other HIP sites and precisely the dentate gyrus (GD; -45%), the moderately lower NR1 levels seemed to go in an opposite direction to those of the euthermics (check Figure 4E). Regarding AMPAR subtypes, CPP-treated hamsters displayed both strong and moderately reduced GluR1 mRNA levels in the other AMY areas, i.e. the central nucleus (Ce, -70%) and in Or-Py (-52%), respectively (Figure 5B). In contrast, GluR2 exhibited a net up-regulatory pattern in mostly HTH areas as demonstrated by the strong increase in the arcuate nuclues (Arc, +74%).

**Figure 6:**
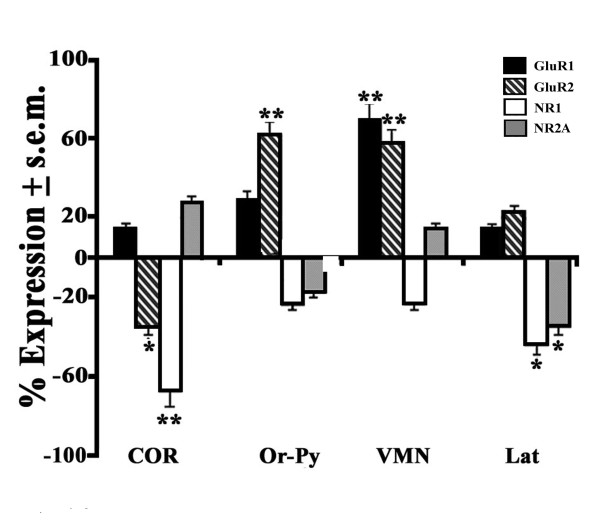
**Brain expression of AMPAR and NMDAR mRNAs in CNQX-treated euthermics**. Transcript values derived from similarly defined sections to those of CPP treatment in Figure 4. Differences of GluR1 (black), GluR2 (black-white stripes), NR1 (white) and NR2A (grey) mRNAs of CNQX-treated hamsters (n = 4) were compared to their controls (n = 4) and expressed as a percentage (%) ± s.e.m. For CNQX-induced statistical variations on AMPAR and NMDAR mRNAs in euthermic hamsters plus abbreviations check Figure 4.

**Figure 7:**
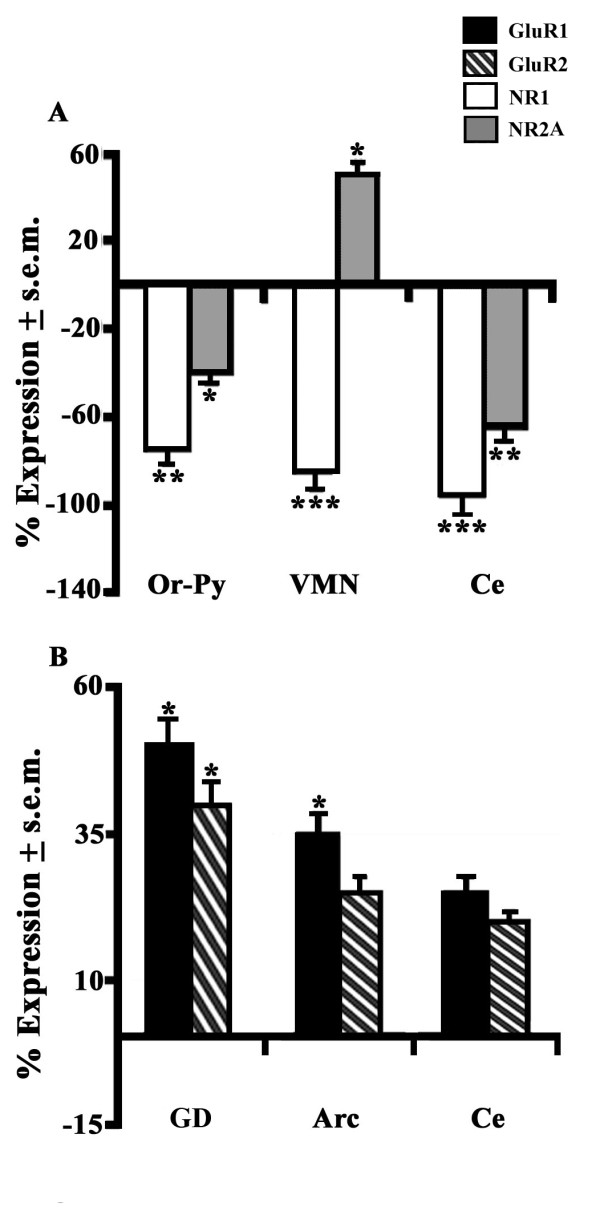
**Brain expression levels of NMDAR and AMPAR on CNQX-treated hamsters during torpor state**. The effect of CNQX (n = 4) on the expression percentage (% ± s.e.m.) of (A) NR1 (white) and NR2A (grey) plus of (B) GluR1 (black) and GluR2 (black-white stripes) in some telencephalic and HTH areas of hibernators was compared to controls (n = 4). For CNQX-induced statistical variations on AMPAR and NMDAR mRNAs of hamsters during torpor state plus abbreviations check Figure 4.

Curiously, in spite of the limited behavioral effects induced by CNQX, this AMPAR antagonist provided consistently greater expression patterns of both classes of Glu subtypes in the different limbic areas. First of all, the AMPAR antagonist accounted mostly for a great up-regulatory type of effect (+58 to +70%) on GluR1- (VMN) and GluR2- (VMN, Or-Py) expressing neurons of euthermics versus controls while a moderate down-regulation was instead reported for COR GluR2 levels (-38%) under these same conditions (Figure 6). Conversely, this same treatment induced a down-regulatory type of effect on NMDAR transcripts as shown by great NR1 levels in COR (-68%) and moderately reduced NR1 (-47%) and NR2A (-35%) levels in Lat. Of particular interest is the role of CNQX on the expression ability of the same brain areas of hibernating hamsters. This is mostly evident for NR1-expressing neurons that displayed a very great down-regulation of NR1 in Ce (-95%) and VMN (-85%), while greatly reduced levels were detected for Or-Py NR1 (-71%) and for NR2A of this same AMY nucleus (-65%) levels (Figure 7A). In the case of AMPAR-expressing neurons, CNQX exerted an up-regulatory type of activity as shown by great GluR1 levels in GD (+50%) and moderate levels in Arc (+37%) whereas GluR2-expressing neurons only showed moderately high levels (+40%) in the former brain area of hibernating hamsters (Figure 7B).

## Discussion

In this first study, the prevalent role of AMPAR and NMDAR subtypes on drinking behavior turned out to be a determining driving stimulus for the arousal state of our hibernating hamster model. Previous works have shown that ambient conditions like photoperiod, temperature and food shortage are among the major factors regulating the transition from euthermic to torpor state and then to arousal of permissive hibernators [29,33]. Dense glutamate receptors levels in the different AMY subnuclei, above all in BLA complex, plus HIP and HTH sites corroborate the importance of glutamatergic neuroreceptors in hibernating rodents [34]. Hence, while the infusion of BLA with NMDAR antagonist tends to strongly favor increased drinking bouts, it seems to account for less motor activity especially during the last two days of hibernation that coincide with arousal state. As a consequence, it appears that the main NMDAR actions of limbic areas during hibernation are aimed at satisfying some of the major physiological states via glutamatergic neuronal mechanisms [22,35]. The fact that elevated drinking frequencies resulted in euthermics and above all in hibernators following infusion of BLA with CPP point to this AMY NMDAR site as a favorable target for such a vital function. This relationship is in line with lesions AMY failing to increase feeding bouts, which strengthens the failure of hamsters to display any eating stimuli aside prior to awakening state (6^th^-7^th ^day) in which CPP-treated hibernators displayed moderately significant increases of feeding intervals [36]. Moreover, such a relationship tends to fit well with HTH stressors accounting for blocked hunger stimuli despite the presence of food sources and so hibernators do not show any desire of eating during torpor while such an action is deactivated during the arousal state [37]. In this context, projections of BLA glutamatergic fibers towards the lateral HTH area, which form part of the historical feeding circuit linked to initiation of eating bouts, suggests that its topographically pivotal position exerts a key switching role not only for hunger state, but above all for drinking rhythms during arousal phase. Thus the induction of drinking behavior in hibernators by NMDAR signals may constitute a first major BLA stimuli responsible for the release of osmotic factors directly or indirectly via the activation, among other, of vasopressin [1,38,39] that could in turn lead to the promotion of the arousal state in hamsters.

We next checked to establish which specific BLA glutamatergic subtype(s) was involved with increased drinking rates. From the altered NMDAR expression levels of CPP-treated hamsters accounting for modified drinking bouts, it seems that this subtype in various limbic areas is responsible for such modifications. A strong up-regulation of NR1 mRNA levels was reported for telencephalic areas such as COR of euthermics while a strong down-regulation of NR2A levels characterized both this brain area and AMY of non-hibernating hamsters. In the case of hibernators, a moderate down-regulation was instead registered for VMN NR1 levels, while an inverted pattern was typical of the AMY and HIP. These results appear to support a NR1- and NR2A/2B-dependent facilitation of drinking motivation, which is in agreement with enhanced licking motivational stimuli being related to a rapid activity-dependent redistribution of HTH synaptic and extra-synaptic NMDAR domains [39]. Interestingly, such a feature seems to rely on the cross-talking of BLA with other regions as shown by the infusion of this AMY complex with NMDAR antagonists evoking increased motor behaviors plus a reduction of feeding bouts probably via interactions with other telencephalic areas like the nucleus accumbens, which controls food seeking and intake [40,41].

It is worthy to note that while the disruption of the tonic inhibitory component of BLA excitatory NMDAR mechanisms seems to preferentially control drinking rhythms, AMPAR signals appeared to be instead responsible for the induction of exploratory type of locomotor behaviors in our hibernating rodent [42,43]. This condition tends to be confirmed, aside by HTH AMPAR neuronal cues accounting mostly for the induction of feeding behaviors and consequently a greater body mass in hibernators, by CNQX in other brain areas like COR and HIP promoting exploratory type of behaviors [44-47]. Now, the fact that neuronal NMDAR activities modify drinking frequencies, especially during hibernating bouts should be of no surprise since moderately significant down-regulated NR1 expressing neurons were typical of key HTH areas such as VMN while a strong reduction of GluR1 mRNAs characterized Ce and Or-Py. Consequently, CPP-dependent behavior and neuronal expression relationships appear to be attributed to the blocking of exploratory stimulus via the inhibition of silent neurons (GluR1 and NR1), at least during hibernation. Conversely, their activation during arousal might serve as a driving stimulus for the promotion of exploratory activities [48]. Indeed altered brain water homeostasis during this physiological state, leading to the shrinkage of cerebral cells seem to be typical of hypoxic metabolic processes and so may very well contribute, along with the inhibition of GluR1 and NR1 activities to the triggering of hyperphagic and drinking responses prior to arousal [25,49,50]. Furthermore, a synergic interaction of NMDAR and AMPAR sites during the ischemic episodes of arousal favorably support plasticity events via an interaction of NR1/2B with GluR1-dependent phosphorylative mechanisms that have been recently proposed for their therapeutic values against white matter diseases [51]. On the other hand such a feature may be also tightly linked to the influences of other neuronal systems and namely the GABAergic system since interneuron signaling of a vast number of encephalic fields are involved with GABA_A_R-related glutamatergic plasticity events [52] and this type of relationship may constitute interesting future research prospects.

## Conclusions

The results of present study have shown for the first time that glutamatergic neuroreceptor system to play a key role on vital physiological activities of the facultative hibernating golden hamster during the different states of hibernation. In particular, it seems that the down regulatory activities of NR1/2A account for the activation of the arousal state through drinking stimuli, which are in line with the influence of NMDAR excitatory system towards the promotion of the different hibernating states in the Arctic ground squirrel [15]. Curiously, it was glutamatergic cross-talking activities of BLA eliciting drinking and not eating stimuli that resulted to be a very strong switching "on" mechanism for the induction of the arousal state. Now, because ischemic risks are at a peak in the waking state of hamsters due to very low body food storages, like in the arousal thermogenesis phase, makes this permissive facultative hibernating rodent a good model for studying neurodegenerative and neuroprotection events during such a critical physiological state [22,32,51].

## Abbreviations

Glu: glutamate; CPP: 3-(+)-2-carboxypiperazin-4-yl-propyl-1-phosphonate; NMDAR: N-methyl-D-aspartate receptor; CNQX: cyano-7-nitro-quinoxaline-2,3-dione; AMPAR: acid α-amino-3-idrossi-5-metil-4-isoxazol-propionic receptor; BLA: basolateral complexes; GluR1: glutamatergic type 1 receptor; GluR2: glutamatergic type 2 receptor; VMN: ventromedial hypothalamic nucleus; NR1, NR2A-D, NR3: N-methyl-D-aspartate receptor subtypes; REM: rapid eye movement; DEPC: diethylpyrocarbonate; RT: Reverse transcription; PCR: Polymerase chain reaction; DIG: digoxigenin-11-dUTP; EDTA: ethylene diamine tetraacetic acid; PBS: phosphate buffer solution; NBT/BCIP: alkaline phosphatase color reaction buffer; ANOVA: Analysis Of Variance; COR: cortex; Or-Py: hippocampal oriens-pyramidalis layer; Lat: lateral amygdala nucleus; VMN: hypothalamic ventromedial nucleus; GD: hippocampal dentate gyrus; Ce: amygdalar central nucleus; Arc: arcuate nucleus.

## Competing interests

The authors declare that they have no competing interests.

## Authors' contributions

RA and MC conceived, designed the experiments, developed and performed RT-PCR experiments, wrote and edited the manuscript. AE, CA and FRM handled *in situ *hybridization and RT-PCR and performed the statistical analysis plus helpful editing contributions. All authors participated in analysis of dates, read and approved the final manuscript.
